# Scalp‐recorded N40 visual evoked potential: Sensory and attentional properties

**DOI:** 10.1111/ejn.15443

**Published:** 2021-09-27

**Authors:** Alice Mado Proverbio, Veronica Broido, Francesco De Benedetto, Alberto Zani

**Affiliations:** ^1^ Department of Psychology University of Milano‐Bicocca Milan Italy; ^2^ Milan Center for Neuroscience (NeuroMi) University of Milano‐Bicocca Milan Italy; ^3^ School of Psychology Vita Salute San Raffaele University Milan Italy

**Keywords:** attention, EEG/ERPs, gratings, LGN, spatial frequency, thalamus, VEP

## Abstract

N40 is a well‐known component of evoked potentials with respect to the auditory and somatosensory modality but not much recognized with regard to the visual modality. To be detected with event‐related potentials (ERPs), it requires an optimal signal‐to‐noise ratio. To investigate the nature of visual N40, we recorded EEG/ERP signals from 20 participants. Each of them was presented with 1800 spatial frequency gratings of 0.75, 1.5, 3 and 6 c/deg. Data were collected from 128 sites while participants were engaged in both passive viewing and attention conditions. N40 (30–55 ms) was modulated by alertness and selective attention; in fact, it was larger to targets than irrelevant and passively viewed spatial frequency gratings. Its strongest intracranial sources were the bilateral thalamic nuclei of pulvinar, according to swLORETA. The active network included precuneus, insula and inferior parietal lobule. An N80 component (60–90 ms) was also identified, which was larger to targets than irrelevant/passive stimuli and more negative to high than low spatial frequencies. In contrast, N40 was not sensitive to spatial frequency per se, nor did it show a polarity inversion as a function of spatial frequency. Attention, alertness and spatial frequency effects were also found for the later components P1, N2 and P300. The attentional effects increased in magnitude over time. The data showed that ERPs can pick up the earliest synchronized activity, deriving in part from thalamic nuclei, before the visual information has actually reached the occipital cortex.

## Graphical abstract

Recorded for the first time on the human scalp electrical signals deriving from the thalamus 40 ms after the onset of visual stimulation. The EEG data showed that visual evoked potentials (VEPs) can detect signals that originate from subcortical structures before visual information has actually reached the occipital cortex. These findings indicate that the thalamus not only transmits sensory signals but also acts as an attentional filter. This paper describes sensory and attentional properties of newly defined N40 component of VEPs.

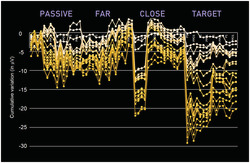

**Abbreviations**
ANOVA: analysis of variance
BA: Brodmann area
C/deg: cycles per degree
CC: cingulate gyrus
EEG: electroencephalogram
EOG: electro‐oculogram
ERP: event‐related potential
FG: fusiform gyrus
ISI: inter‐stimulus interval
ITG: inferior temporal gyrus
LGN: lateral geniculate nucleus
μV: microvolt
MFG: medial frontal gyrus
MOG: middle occipital gyrus
MTG: middle temporal gyrus
nA: nanoampere
RT: reaction time
SD: standard deviation
SE: standard error
SFG: superior frontal gyrus
STG: superior temporal gyrus
SPL: superior parietal lobule
SwLORETA: standardized weighted low‐resolution electromagnetic tomography
TRN: thalamic reticular nucleus

## 1 INTRODUCTION

Although the C1 (or P/N80) component of visual evoked potentials (VEPs) has been known for some time and has been extensively studied by electrophysiologists for its sensory and attentional characteristics (Bodis‐Wollner et al., 1992; Capilla et al., 2016; Clark et al., 1995; Jeffreys & Axford, 1972; Proverbio et al., 2010; Proverbio, Del Zotto, & Zani, 2007; Regan, 1989; Zani & Proverbio, 2009, 2018, 2020; Zhang et al., 2015), existence itself of an N40 thalamic visual potential recorded by the scalp is still a matter of debate. N40 is a rather well‐known component with respect to the auditory (e.g. Adler et al., 1982) and somatosensory (e.g. Allison et al., 1989) channels but not much recognized as regards the visual one. Much evidence for the existence of N40 derives from subcortical recordings, which exhibit high spatial resolution and derive their signals from intracranial electrodes implanted in animals (e.g. Schroeder et al., 1989, 1992) or in patients with grids of implanted electrodes or during stereotaxic surgery (e.g. Choi et al., 1977). It was first identified, using deep recording techniques, in response to flash stimulation in primates (Kraut et al., 1985, 1990).

Neurophysiological recordings using multichannel electrodes have shown that lamina 4C in the macaque contributes to the surface flash‐VEP N40 (Kraut et al., 1985; Schroeder et al., 1992) and to the pattern VEP N40 (Schroeder et al., 1991). Moreover, Tenke et al. (1993) reported that the main contribution to the initial scalp‐recorded N40 of the VEP in monkey is generated by a combination of presynaptic activation of the axon terminals of the thalamo‐cortical afferents and of excitatory presynaptic potentials on the stellate cells within lamina 4C.

In an interesting study, Givre et al. (1994) recorded surface VEPs as well as multiunit activity evoked by light flashes from V1 and V4 areas of three alert macaque monkeys. They found that V1 mostly contributed to N40 and P55–80 component, whereas V4 mostly contributed to later N95, P120 and late negativity responses. V4 was also partially involved in N40 generation as an afferent‐triggered inhibition bypassing V1, according to the authors. Unfortunately, thalamic activity was not recorded directly in these studies, so the extent of thalamic contribution to N40 is not known. As stated by the authors themselves, subcortical structures including the pulvinar (e.g. Tanaka et al., 1990) and the interlaminar cells in the lateral geniculate nucleus (LGN) (e.g. Yoshida & Benevento, 1981) project directly to V4. Therefore, it is possible that the early N40 activity seen in V4 (prior to the arrival of afferents from supragranular V1) would reflect fast lateral connections to the thalamic nuclei. One of the main contributions of this study is that it demonstrated a close correlation between VEPs recorded on the scalp and inner intracranial potentials (see also Schroeder et al., 1992). As for the timing of sensory components measured intra‐cortically, according to Kelly et al. (2010), a 3/5th‐scaling rule should be used for comparing simian versus human timing so that a given latency difference measured in non‐human primates should be scaled up by this factor. It is therefore possible that human magnocellular P40 (Vaughan, 1966) elicited by light flashes (P34 potential in Harding & Rubinstein, 1980, 1982) and parvocellular N40 elicited by spatial frequency gratings (in this study) correspond to simian N25 potential in terms of latency. However, as argued by Ales et al. (2013), some of the reported differences across human and simian studies might also depend on methodological factors, such as differences in stimulus size. For example, Schroeder et al. (1989, 1991) stimulated monkeys with a very large flash of light subtending 20° and recorded a V1 response at 26 ms of latency. Conversely, Clark et al. (1995) used a much smaller chequerboard pattern and recorded a C1 response in humans at 40–45 ms of latency. It is not easy to exactly determine the role of stimulus size in modulating the latency of sensory responses across human and non‐human primates. For example, in another study performed in humans by Farrell et al. (2007) using subdural electrodes placed on calcarine cortex, it was found that V1 response to pattern reversal stimuli was about 45–55 ms, which roughly corresponds to the macaque's latency (Ales et al., 2013).

The other problem is finding a correspondence between scalp‐recorded and intracranial potentials. At this regard, Kraut et al. (1985) recorded flash VEPs in monkeys both from the cortical surface and intracortically to find a correspondence between intracranial potentials and surface potentials. They found that the two subsequent surface‐negative potentials N25 and N40 were generated within laminae IVA and IVCb, respectively, both parvocellular thalamo‐recipient layers. In summary, both N25 and N40 simian potentials would be generated mainly by the synaptic thalamo‐cortical excitatory inputs in lamina IV (see Kraut et al., 1985).

Relative to human studies, Pratt et al. (1982) recorded flash evoked potentials within the first 100 ms following photic stimulation and concluded that the earliest potentials were generated in the optic nerve or tracts, whereas the later components in thalamo‐cortical structures. Later, Pratt et al. (1995) found scalp‐recorded potentials generated by subcortical structures along the visual pathways, including the optical nerve. Harding and Rubinstein (1980, 1982) measured the flash evoked subcortical potential (VESP) of mean latency P23–N28–P34 and hypothesized that their neural generators might be entirely subcortical and topographically separate from the lid electroretinogram, on one side, and the visual evoked cortical potential, on the other side. The triphasic wave showed a centro‐parietal distribution, slightly posterior to the Rolandic/Sylvian fissure. Again, Kraut et al. (1990) found that the first significant VEP component, N40, was generated principally within the parvocellular thalamo‐recipient sublamina 4Cb. In fact, N40 is now believed to reflect early brain activity caused by activation of the LGN of the thalamus.

The issue of whether this inner electrical activity might be detected at scalp surface has been discussed more recently. Attal and Schwartz (2013) measured alpha power modulations in a group of seven healthy subjects (by contrasting the closed‐eyes with open‐eyes conditions) with MEG and applied several source reconstruction techniques to identify the intracranial sources of electromagnetic activity. They found that subcortical activity and particularly thalamic activations could be reliably detected, notwithstanding thalamus is mainly composed of stellate cells (closed fields) and its neural density is estimated to be 10 times lower than that of neocortex, thus producing smaller neural currents. In details, they found that sLORETA was able to detect thalamic sources with a dipole localization error (DLE) under 0.5 cm. Other EEG studies have shown a significant overlap between scalp‐recorded EEG signals and intracranial EEG signals with respect to the localization of the source (Mégevand et al., 2014). In fact, the images extracted from high‐density EEG recordings source imaging seem to have 85% accurate localization capability (Brodbeck et al., 2011). However, this spatial resolution can be achieved only if individual MRI‐derived head models are used. One controversy concerns the question of whether surface EEG can detect subcortically originating postsynaptic potentials. Indeed, Seeber et al. (2019) with high‐density recordings (256‐channel) showed that the alpha activity (8–10 Hz) recorded by the scalp was highly comparable with alpha recorded by the electrodes implanted in the medial nucleus of the thalamus. They investigated whether scalp EEG might detect and localize EEG signals recorded with intracranial electrodes located in the centro‐medial thalamus, as well as in the accumbens nucleus in three patients, during eyes closed relaxation. It found a strong correlation between alpha envelopes derived from intracranial and EEG source reconstructed signals. These evidences show that scalp EEG can indeed detect subcortical signals, as also shown by previous studies (Harding & Rubinstein, 1980; Pratt et al., 1982; Pratt et al., 1995; Pratt et al., 2000; Schroeder et al., 1992).

**FIGURE 1:**
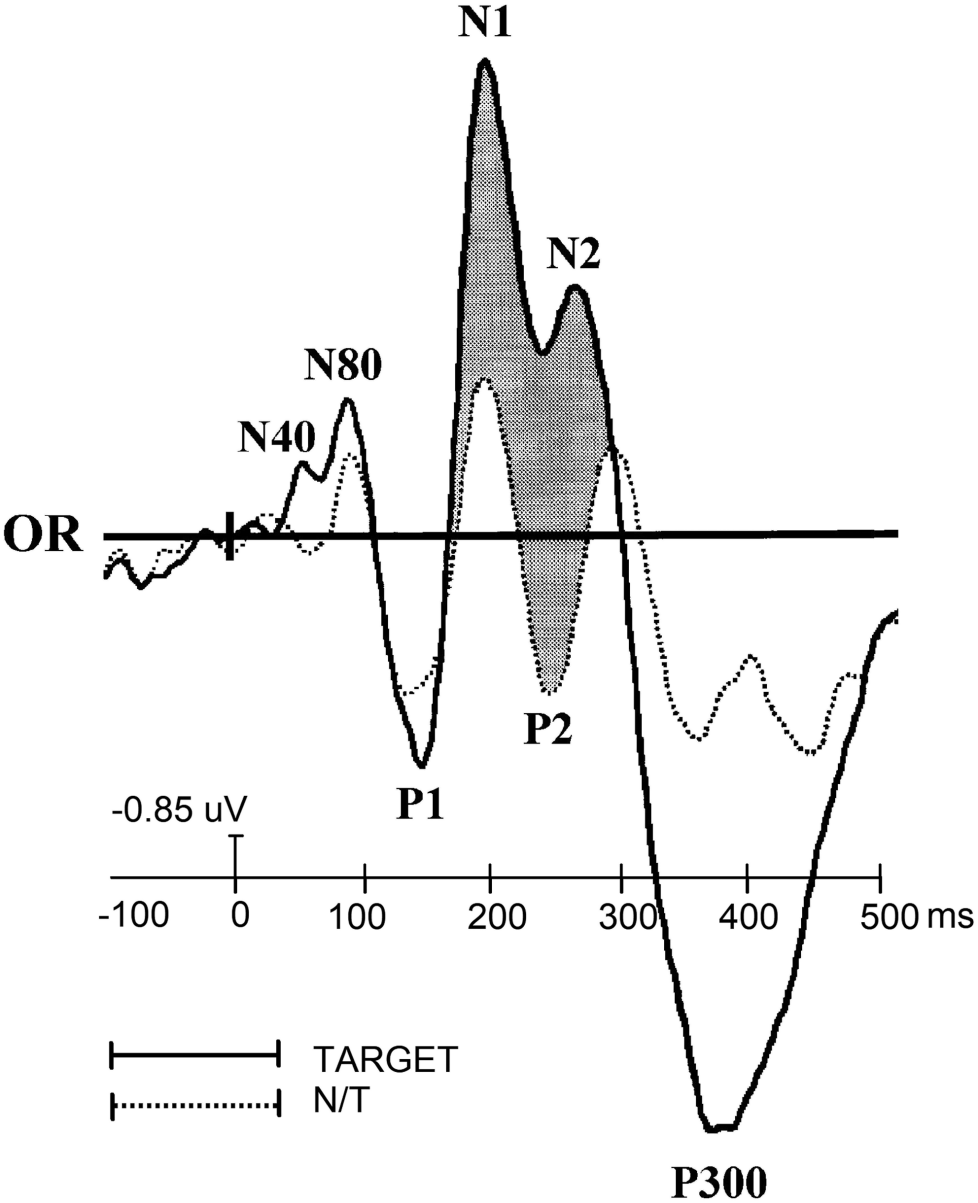
Grand‐average ERPs recorded at the right lateral occipital electrode in response to foveally presented gratings of 6 c/deg during a task of selective attention to spatial frequency. (Taken from Proverbio & Zani, 2002 [figure 7 of chapter 2, p. 28] with permission of the editors, authors and publisher)

Overall, the N40 recorded on the scalp is likely to partly reflect activity of subcortical nuclei, such as pulvinar and thalamus (Givre et al., 1994; Schroeder et al., 1992). The first description of a scalp‐recorded N40 visual response in a psychophysiology handbook dates back to 2002 (Proverbio & Zani, 2002), where it is also reported that it appears to be modulated by attention to spatial frequency gratings (see Figure 1).

The aim of the present study was to gain further knowledge on the sensory and attentional property of N40 component of scalp‐recorded VEP/ERPs in a passive viewing and attentional task. In detail, we aimed to assess whether N40 was sensitive to stimulus spatial frequency and modulated by attention. In fact, neuroimaging and neurophysiological studies have provided evidence of a sensitivity of LGN and pulvinar nuclei of thalamus to attentional allocation (Bender & Youakim, 2001; Halassa & Kastner, 2017; Kastner & Pinsk, 2004; McAlonan et al., 2006, 2008; Saalmann & Kastner, 2014; Schneider, 2011; Vanduffel et al., 2000; Wimmer et al., 2015). For example, McAlonan et al. (2008) recorded from LGN and thalamic reticular nucleus (TRN) neurons in attending macaque monkeys (*Macaca mulatta*) and found that attention modulated visual signals before they even reached V1 cortex by increasing responses of both magnocellular and parvocellular neurons in LGN and decreasing neuronal responses in the adjacent inhibitory TRN neurons. Again, in a fMRI study in which human subjects covertly directed attention to a chequerboard arc to detect randomly occurring luminance changes (O'Connor et al., 2002), it was found that not only the LGN enhanced neural responses to attended stimuli but also inhibited neural responses to unattended stimuli while increasing baseline activity in the absence of visual stimulation. The authors concluded that thalamus served as early gatekeeper in controlling attentional response. Here, it was investigated whether thalamus was possibly modulated by attention (by comparing N40 elicited by targets vs. non‐targets) or by arousal (by comparing N40 to passively viewed vs. attended [or unattended] stimuli).

Although the electrophysiological literature has clearly shown a P/N80 modulation of striate C1 visual evoked response due to attentional selection of gratings or check size patterns (e.g. Capilla et al., 2016; Proverbio et al., 2010; Proverbio, Del Zotto, & Zani, 2007; Zani & Proverbio, 2018; Zhang et al., 2015), the evidences of an earlier N40 attentional effect are very scant. We hypothesized that if the N40 was sensitive to increased attention or alertness, we should have observed an increase in N40 negativity towards targets compared with non‐target or passively viewed gratings, as we actually did.

In the present study, we expected to find well‐known attentional modulations at P/N80, P1, N2 and P300 level, with larger amplitude to target than non‐target, and to non‐target than passively viewed gratings (e.g. Kenemans et al., 1993; Koivisto & Revonsuo, 2008; Martínez et al., 2001; Proverbio et al., 2002; Zani & Proverbio, 2012). Furthermore, an effect of spatial frequency on the polarity of N80 was expected to be found, with larger N80 responses to high spatial frequencies and larger P80 responses (or smaller negativities) to low spatial frequency gratings (e.g. Bodis‐Wollner et al., 1992; Kelly et al., 2013; Regan, 1989).

## 2 METHODS

### 2.1 Participants

Twenty right‐handed college students (10 males, 10 females) with normal or lens‐corrected vision took part in the study. One subject was discarded after EEG recording due to an insufficiently accurate performance. The remaining group consisted of 10 males (average age: 22.4) and nine females (average age: 21.9). All participants were psychically and neurologically healthy. Prior to EEG recording, participants were asked to complete research informed consent and minimal risk documentation. They were then administered the Edinburgh Inventory to assess their right‐handedness. Experiments were conducted with the understanding and written consent of each participant according to the Declaration of Helsinki (BMJ 1991; 302: 1194), with approval from the Ethical Committee of the University of Milan‐Bicocca (Prot. RM‐2019‐177).

### 2.2 Stimuli

**FIGURE 2:**
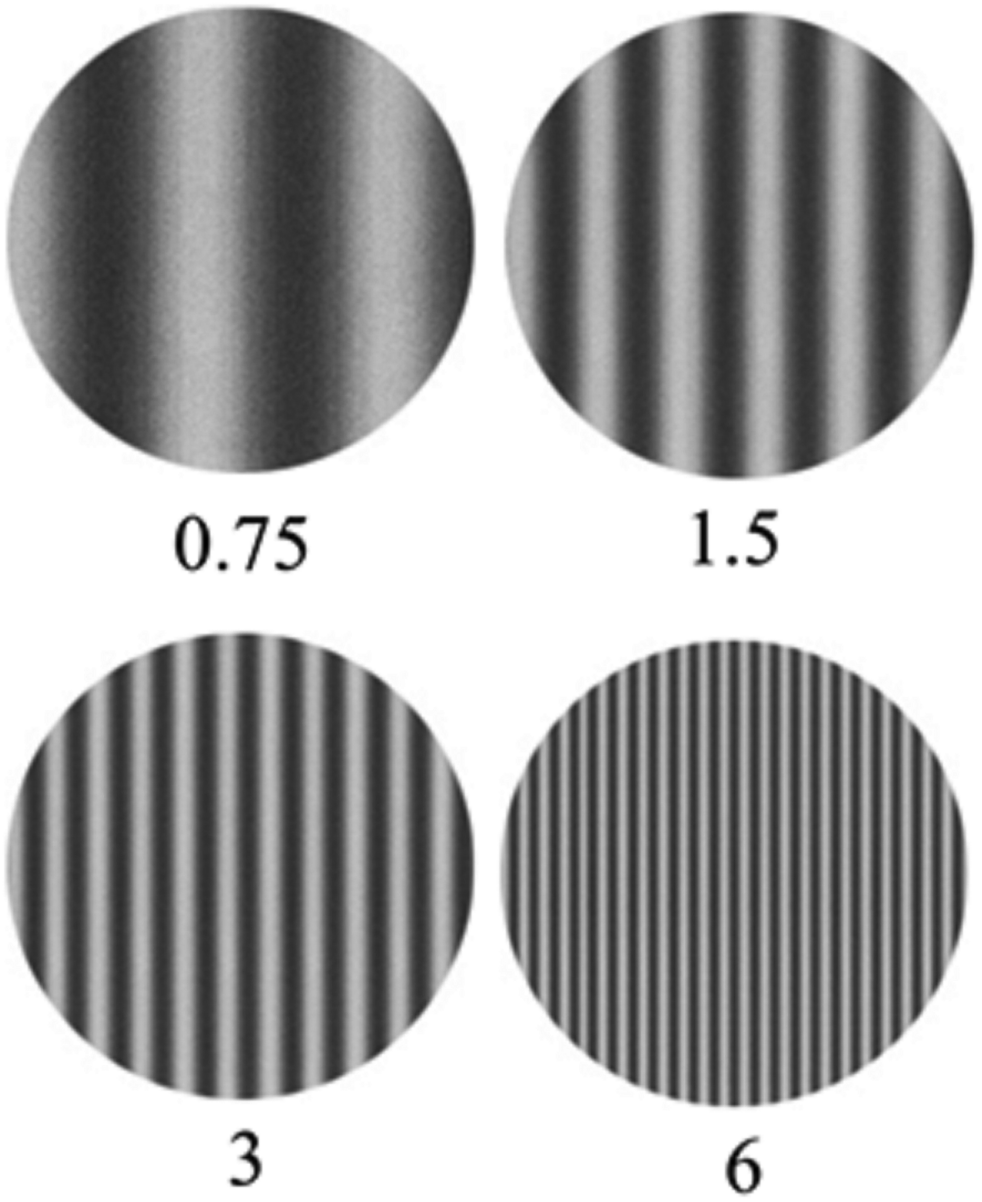
Vertical sinewave gratings used in the study. The number indicates their spatial frequency in cycles per degrees (c/deg)

The stimuli were four vertical black and white sinusoidal gratings. Their spatial frequencies were well visible to the human eye (Maffei, 1978): 0.75, 1.5, 3 and 6 c/deg (Figure 2). Stimuli were presented foveally in pattern‐onset mode for a duration of 80 ms with an inter‐stimulus interval (ISI) of 740 +/− 50 ms (SOA = 770–870 ms). The stimuli were presented on a high‐resolution VGA (Video Graphics Array) screen. The background was grey and isoluminant (average luminance = 35 cd/m^2^; contrast = 40%; brightness = 35%). The gratings had Michelson contrast (C_M_ Lum_max_ − Lum_min_/Lum_max_ + Lum_min_) = 81 and average luminance of 29 cd/m^2^. Gratings had a diameter of 8.2 cm, implying a visual angle of 4°6′.

Stimulus presentation was controlled by *EEvoke* stimulation software (*ANT Software*, Enschede, Netherlands). EEvoke trigger information was sent to the external device for EEG acquisition by means of parallel port connection. EEG signals travelled through bidirectional glass fibres. Inside the stimulation PC, a fibre interface board controlled the glass fibre communication and part of the data processing. Stimulations were controlled through multiple scenarios containing information regarding timing, response pads, event codes and multimedia files. This way, both presentation and corresponding controlling information run in parallel and highly synchronized, thus allowing an optimal synchronization of the stimuli with EEG recordings.

### 2.3 EEG recordings

EEG data were continuously recorded in DC (through ANT Neuro amplifiers) from 128 scalp sites according to the 10–5 International System. Amplifiers features were referential input noise < 1.0 uV rms; referential input signal range = 150–1000 mV pp; input impedance > 1 GOhm; CMRR > 100 dB; max sampling rate = 16.384 Hz across all referential channels; resolution = 24 bit; bandwidth DC (0 Hz)–0.26* sampling frequency.

After A/D conversion, the digitalized EEG was analysed using *EEProbe* recording software (*ANT Software*, Enschede, The Netherlands). Sampling rate was 512 Hz. Horizontal and vertical eye movements were additionally recorded, and linked ears served as the reference lead. Vertical eye movements were recorded using two electrodes placed below and above the right eye, whereas horizontal movements were recorded using electrodes placed at the outer canthi of the eyes, via a bipolar montage. The EEG and electro‐oculogram (EOG) were filtered with a half‐amplitude bandpass of 0.016–70 Hz. Filter features were offline finite impulse response (FIR), non‐causal, symmetric, linear phase response, using a Hamming window (standard coefficients 0.54 and 0.46). Filter order/length = 701 points. Electrodes impedance was maintained below 5 KOhm. EEG epochs were synchronized with the onset of grating presentation and analysed using ANT *EEProbe* software. Computerized artefact rejection was performed prior to averaging to discard epochs in which amplifiers blocking, eye movements, blinks or excessive muscle potentials occurred. The artefact rejection criterion was a peak‐to‐peak amplitude exceeding 50 μV and resulted in a rejection rate of ∼10%. Artefact rejection rates for the different conditions were ‘near’ = 11.95%, ‘target’ = 9.31%, ‘passive’ = 6.81%, ‘far’ = 10.14%; min = 2.5%, max = 27.5%.

### 2.4 Procedure

The participants were seated in a faradized and anechoic cubicle and were instructed to fixate the centre of a screen located about 114 cm from their eyes, to relax, not to contract face or body muscles and to avoid blinking as much as possible.

The participant's task was to press a key with the index finger of the left or right hand (as instructed) in response to the target spatial frequencies, as accurately and quickly as possible. The left and right responding hands were used alternately throughout the recording session. Prior to EEG recording sessions, participants underwent four training sessions of 40 stimuli each in which they familiarized themselves with stimuli, experimental setting and task requirements. The experimental session comprised 15 runs of 120 stimuli each. It included three runs in which target frequency was 0.75 c/deg, three runs in which target frequency was 1.5 c/deg, three runs in which target frequency was 3 c/deg, three runs in which target frequency was 6 c/deg and three passive viewing runs. Task conditions were randomly intermixed and counterbalanced across subjects. For the purposes of the ERP averaging, it was considered how close in frequency the non‐targets were to the targets, considering that the width of the spatial frequency channels (Harter & Previc, 1978; Maffei, 1978; Zani & Proverbio, 1995) is about 1 octave. Therefore, the non‐targets were subdivided into ‘close’ to and ‘far’ from target frequency (see Table 1).

**TABLE 1:** Experimental conditions and relative stimulus categories

Stimulus	Attentional condition				
c/deg	0.75	1.5	3	6	No target
0.75	Target	Close	Far	Far	Passive
1.5	Close	Target	‐	‐	Passive
3	‐	‐	Target	Close	Passive
6	Far	Far	Close	Target	Passive

*Notes*: Each target spatial frequency was compared with the closest spatial frequency, therefore slightly task irrelevant, and to the farthest spatial frequency, therefore strongly irrelevant for the task. The passive viewing condition, in which the general alertness level was lower, was used as a baseline condition to appreciate the effect of attentional and alertness allocation on ERP components. Evoked responses were recorded to the same physical stimulus and compared across attentional conditions. For example, ERPs elicited by 1.5 c/deg gratings when targets were compared to ERPs elicited by 1.5 c/deg gratings when 0.75 was the target frequency (close N/T) or when 6c/deg was the target frequency (far N/T).

### 2.5 Data analysis

Event‐related potentials (ERPs) were averaged offline from 100 before to 700 ms after stimulus onset. ERP components were identified and measured with respect to the average baseline voltage over the interval from −100 to 0 ms. Isocolour topographic maps of scalp surface voltages were computed in specific time windows. A LORETA (low‐resolution electromagnetic tomography; Pascual‐Marqui et al., 1994) was also applied to surface potentials measured in different time windows and attention conditions, namely, to passive stimuli in the pre‐stimulus −100/−50 ms time window; to passive stimuli between −50 and −0 ms; to the difference signals obtained by subtracting potentials to target minus irrelevant stimuli (close + far) between −55 and −30 ms; to the difference signals obtained by subtracting potentials to target minus passive stimuli between −55 and −30 ms; to passive stimuli in the N40 range (30–55 ms time window); to the difference signals obtained by subtracting potentials to target minus irrelevant stimuli (close + far) in the N40 range (30–55 ms); to the difference signals obtained by subtracting potentials to target minus passive stimuli in the N40 range (30–55 ms); to target stimuli in the N40 time range (+30–55 ms); and to passive stimuli in the 60–90 ms time range. The magnitude of strongest electromagnetic dipoles was compared through Kolmogorov–Smirnov tests (*P* < 0.05) across all conditions (featuring more than four sources).

LORETA, which is a discrete linear solution to the inverse EEG problem, corresponds to the three‐dimensional (3D) distribution of neuronal electric activity that has maximum similarity (i.e. maximum synchronization), in terms of orientation and strength, between neighbouring neuronal populations (represented by adjacent voxels). In this study, an improved version of standardized weighted low‐resolution brain electromagnetic tomography (sLORETA) was used, which incorporates a singular value decomposition‐based lead field weighting: swLORETA (Palmero‐Soler et al., 2007). Source space properties were grid spacing = 5 point, Tikhonov regularization and estimated signal‐to‐noise ratio (SNR) = 3. A realistic boundary element model (BEM) was derived from a T1‐weighted 3D MRI data set by segmentation of the brain tissue. The BEM model consisted of one homogenic compartment made up of 3446 vertices and 6888 triangles (Zanow & Knösche, 2004). The head model was used for intracranial localization of surface potentials. Segmentation and head model generation were performed using the ASA package (ANT Software BV, Enschede, The Netherlands).

The mean area amplitude of N40 response was quantified at P1, P2, PPO1 and PPO2 electrode sites in between 30 and 55 ms. The mean area amplitude of N80 response was quantified at P1, P2, PPO1 and PPO2 electrode sites in between 60 and 90 ms. The electrode choice for peak measurements was based on the previous literature (e.g. Harding and Rubinstein (1980, 1982) for N40 and Zani and Proverbio (2020) for N80 components) and on the scalp topographical distribution of the components during passive viewing conditions.

The mean area amplitude of P1 response was quantified at O1, O2, PPO1 and PPO2 electrode sites in between 90 and 120 ms. The mean area amplitude of N2 component was quantified at P6, P7, PPO10h and PPO9h electrode sites in between 400 and 600 ms. Finally, the mean area amplitude of P300 response was quantified at Cz and Pz electrode sites in between 400 and 600 ms.

Separate four‐way repeated‐measures analyses of variance (ANOVAs) were performed on the amplitude values computed in the various time windows. The factors were ‘spatial frequency’ (0.75, 1.5, 3 and 6 c/deg), attentional condition (target, close, far, passive), ‘electrode’ (dependent on the ERP component of interest) and ‘hemisphere’ (left hemisphere [LH]; right hemisphere [RH]). Post hoc comparisons among means were carried out through HSD Tukey test. We assumed that single *P*‐values near 0.05 provided a weak evidence against the null hypothesis, whereas 0.01 or 0.005 significance values would support stronger evidences.

Response times (RTs) and the percentage of correct responses (hits) were recorded and quantified. RTs that exceeded the mean value ± 2 standard deviations were discarded, which resulted in a rejection rate less than 0.1%. RT data normality was assessed through the Shapiro–Wilk test (Shapiro–Wilk = 0.958). Other generalized linear models could have been more reliable at this aim. For each participant, attention condition (4), spatial frequency (4) and response hand (2), behavioral data were summarized by computing means, corrected means and median values.

Mean RTs and accuracy percentages were subjected to separate multifactorial repeated‐measures ANOVAs with three within‐subjects factors, whose factors of variability were as follows: ‘spatial frequency’ (0.75, 1.5, 3 and 6 c/deg), attentional condition (target, close, far, passive) and response hand (left and right). Accuracy data also underwent non‐parametric tests such as sign test for compensating the lack of normal distribution.

## 3 RESULTS

### 3.1 Behavioral data

**FIGURE 3:**
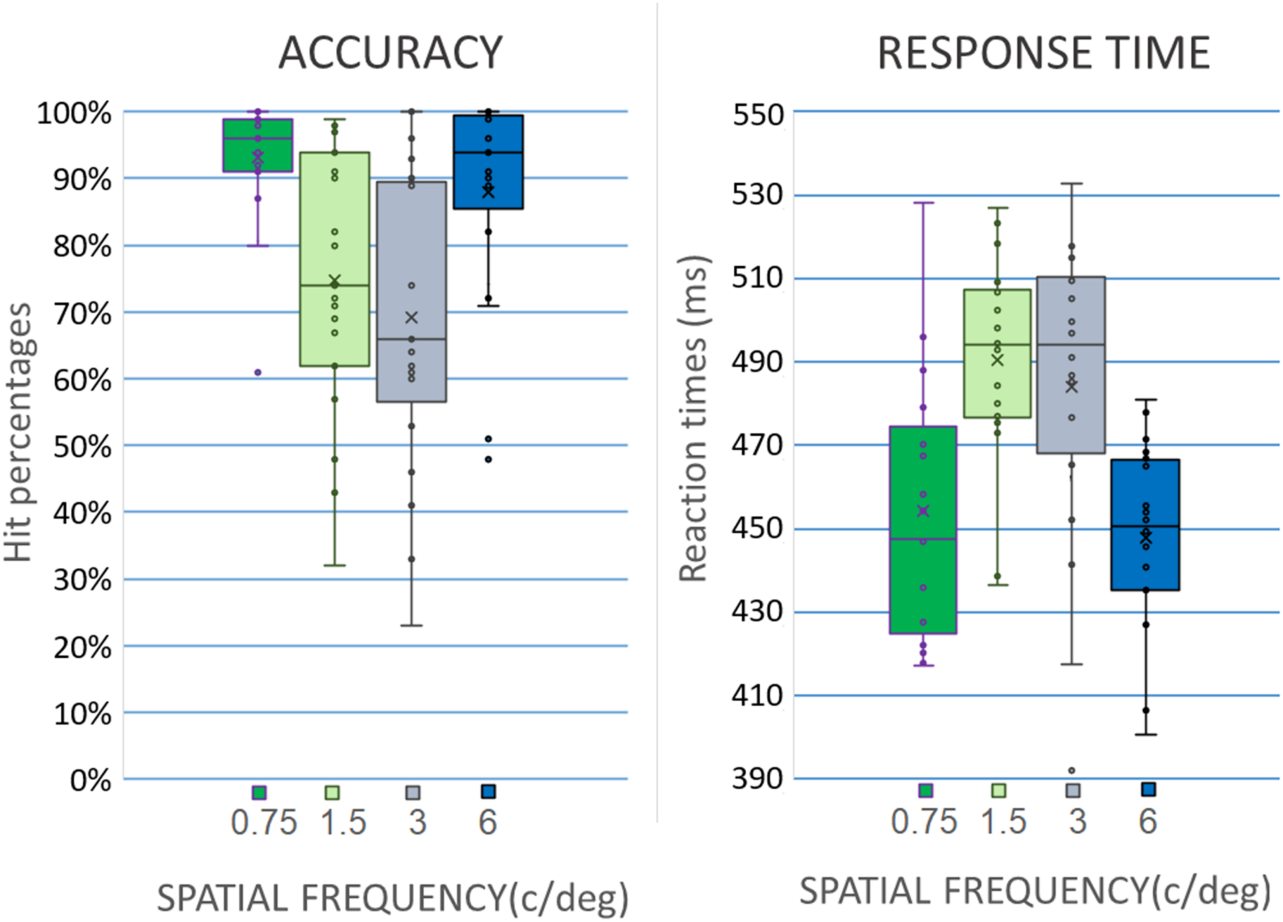
Left: Mean percentages of correct target identifications (hits) as a function of target spatial frequency. The performance was higher for the extreme frequencies: 0.75 and 6 c/deg. Right: Mean response times (RT): Again, RTs were faster for the extreme frequencies: 0.75 (460 ms) and 6 c/deg (454 ms), less prone to interference from neighbouring targets. Scatterplots represent individual data

The accuracy performance of the participants was high overall (hits = 82%, SD = 15), demonstrating that the participants were careful in carrying out the task. Only one participant was excluded from the analysis because he showed an inaccurate performance (hits = 60%). The ANOVA results (*F*(3,54) = 19.34, *P* < 0.0001) showed a better accuracy in the response to 0.75 c/deg (93%, SE = 2.2; confidence interval: −95%/+95% = [88.54; 97.76]) and 6 c/deg (87.89%, SE = 3.7; [80; 95.72]) spatial frequency gratings compared with 1.5 c/deg (74.74%, SE = 4.5; [65.17; 84.29]) and 3 c/deg (69.16%, SE = 5.3; [57.95; 80.36]) spatial frequency gratings (Figure 3, left).

Non‐parametric tests showed identical results. Subjects' performance was similar for frequencies at the boundary of the range (0.75 vs. 6; *P* = 0.422) and much higher than that recorded for intermediate frequencies (0.75 vs. 1.5, *P* < 0.0001; 0.75 vs. 3 c/deg, *P* < 0.0001; 6 vs. 1.5 c/deg, *P* < 0.0001; 6 vs. 3 c/deg, *P* < 0.0001). The performance for intermediate frequencies did not differ statistically (1.5 vs. 3 c/deg; *P* = 0.81).

The average RT of participants was 464.7 ms (SD = 30.7). The ANOVA results (F(3,54) = 7.84, *P* < 0.0001) showed that RTs were faster in response to stimuli with spatial frequency of 0.75 c/deg (459.8 ms, SE = 7.7; [438.5; 467.4]) and 6 c/deg (453.5 ms, SE = 6.6; [437.3; 458.7]) than to stimuli with spatial frequency of 1.5 c/deg (488.1 ms, SE = 9.9; [478.8; 502.1]) and 3 c/deg (484.5 ms, SE = 9.87; [466.7; 501.9]) (Figure 3, right). Moreover, RTs were faster when emitted with the right (464.2 ms, SE = 7.1), compared with the left hand (478.0 ms, SE = 6.5), as shown by significant response hand factor (*F*(1,18) = 8.79, *P* < 0.028).

### 3.2 Electrophysiological data

**FIGURE 4:**
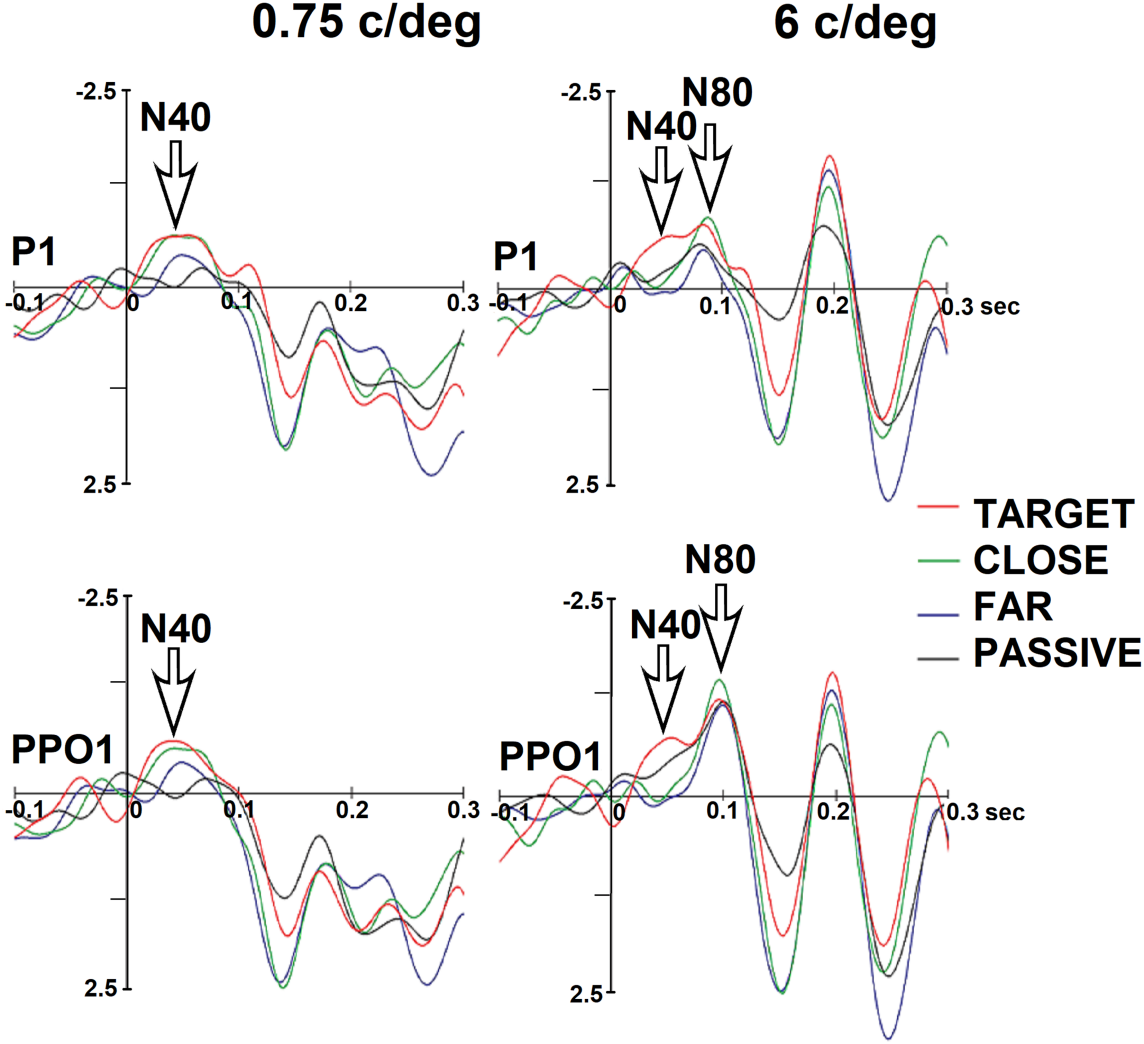
Grand‐average ERPs recorded at left parieto‐occipital (P1) and occipito‐temporal (PPO1) electrodes in response to spatial frequency gratings in the four attention conditions (target, near, far, passive) as a function of stimulus spatial frequency. Please note that voltage is plotted with negative going up

Figure 4 shows grand‐average ERP waveforms recorded at left occipito‐temporal sites in the four attentional conditions (target, close, far, passive) as a function of grating spatial frequency. It can be appreciated the early modulation of both N40 and N80 sensory responses, whose amplitudes were enhanced in response to target gratings.

#### 3.2.1 N40 component (30–55 ms)

**FIGURE 5:**
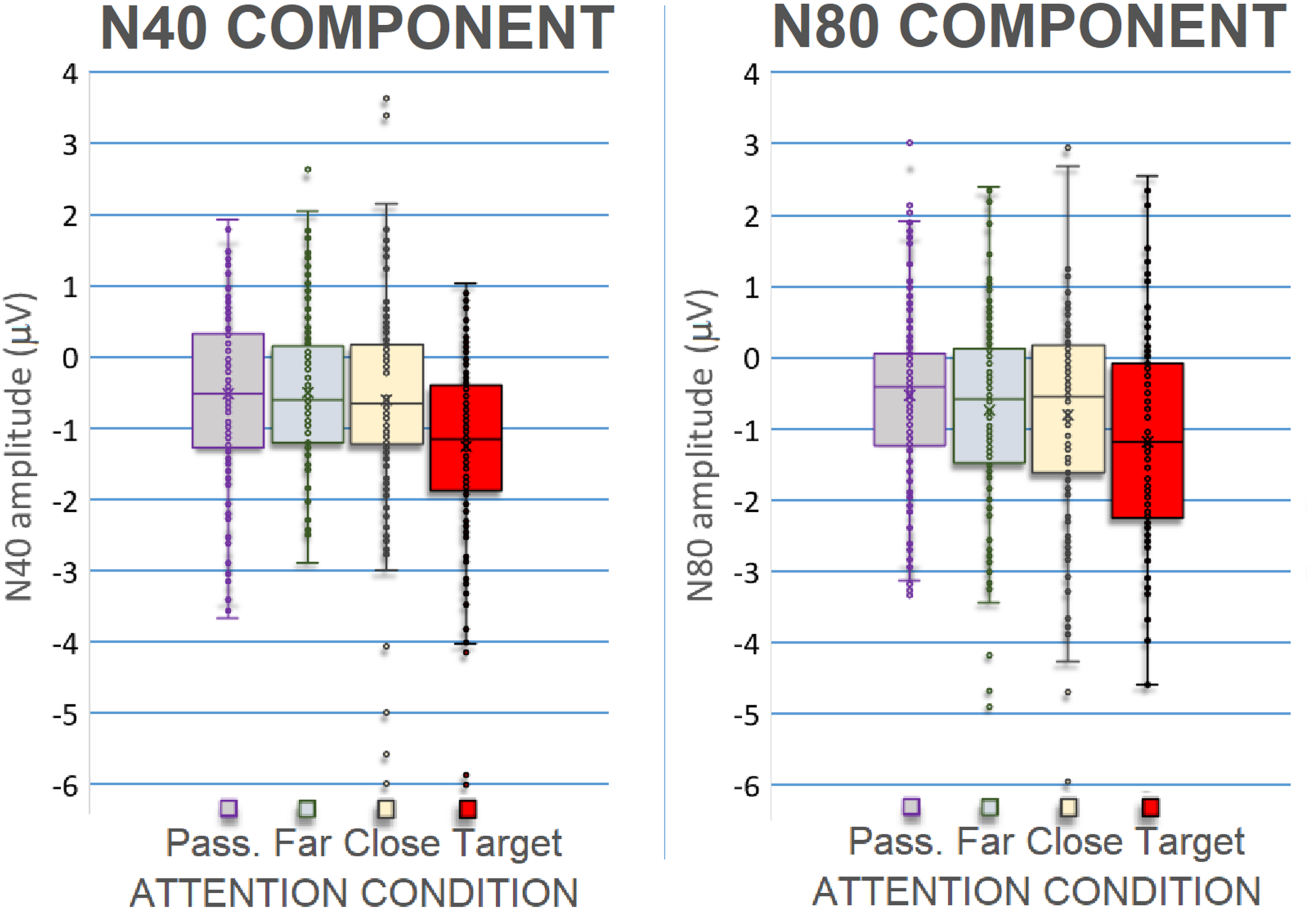
Left: Mean area amplitude values of N40 (30–55 ms) potential recorded at occipito‐parietal sites as a function of attention condition. Scatterplot represents individual data. Right: Mean area amplitude values of N80 potential (60–90 ms) recorded at occipito‐parietal sites as a function of attention condition. N80 response was modulated by selective attention and by alertness. Scatterplot represents individual data

**FIGURE 6:**
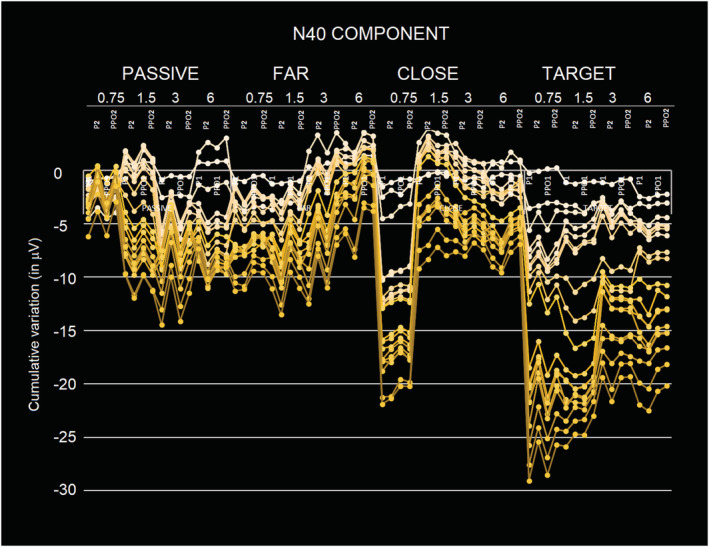
Stacked line charts showing the cumulative variations on N40 amplitude as a function of electrode of recording, cerebral hemisphere, stimulus spatial frequencies and attentional condition. The different traces refer to the different individuals. This figure highlights the strong coherence across individuals of N40 attentive modulation

**FIGURE 7:**
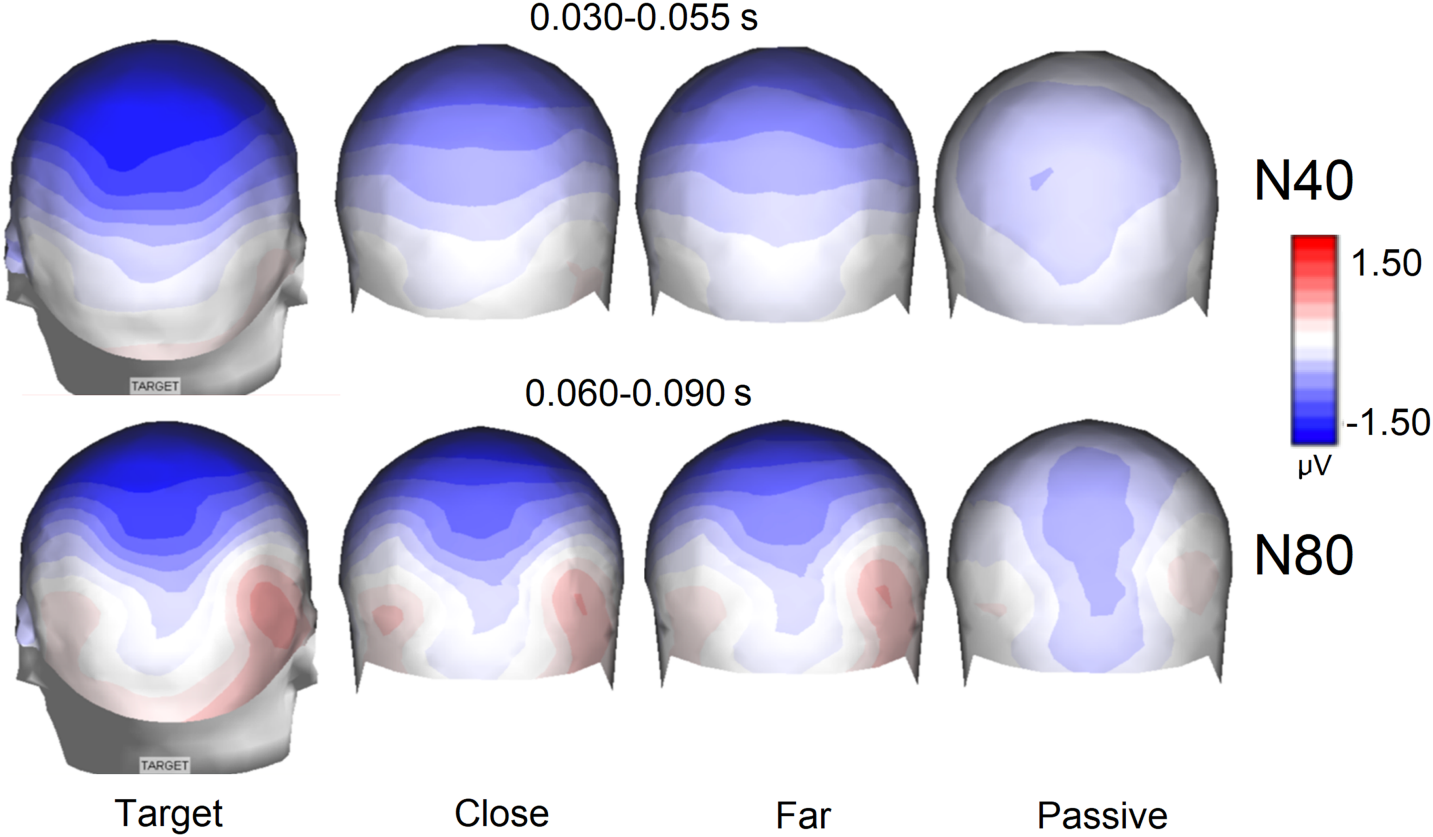
Isocolour topographic maps of the average voltage of the N40 and N80 components recorded in the four attention conditions (target, near, far, passive) regardless of stimulus spatial frequency

The ANOVA performed on the amplitude values of N40 response showed the significance of attention factor (*F*(3,54) = 5.75, p < 0.0017). Post hoc comparisons showed that the N40 elicited by attended stimuli (target = −1.22 μV, SE = 0. 19, [−1.63; 0.82]) was significantly more negative than the N40 elicited by unattended and passively observed stimuli (close = −0.59 μV, SE = 0.24, [−1.087; −0.009]; far = −0.48 μV, SE = 0.15, [0.799; −0.16]; passive = −0.48 μV, SE = 0.13, [−0.769; −0.20]). This effect is well visible in Figure 5 (left) and Figure 6 and in topographical maps of Figure 7.

**FIGURE 8:**
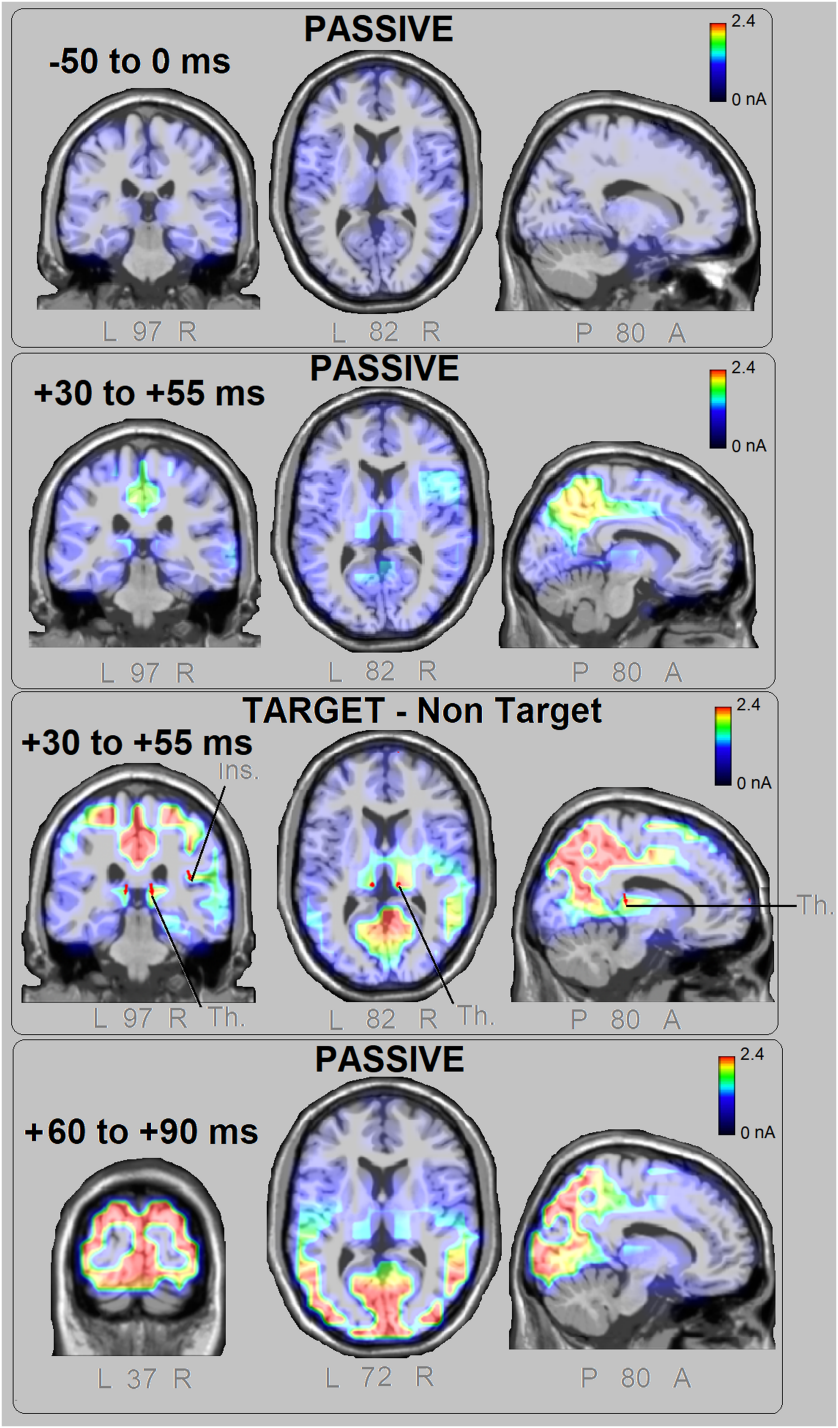
Coronal, axial and sagittal views of swLORETA source reconstruction of surface potentials performed in different pre‐stimulus and post‐stimulus attentional conditions. The various colours represent differences in the magnitude of the electromagnetic signal (nA). The electromagnetic dipoles appear as arrows and indicate the position, orientation and magnitude of the dipole modelling solution applied to the ERP waveform in the specific time window. L, left; R, right; P, posterior; A, anterior; numbers refer to the displayed brain slice in the MRI imaging plane

**FIGURE 9:**
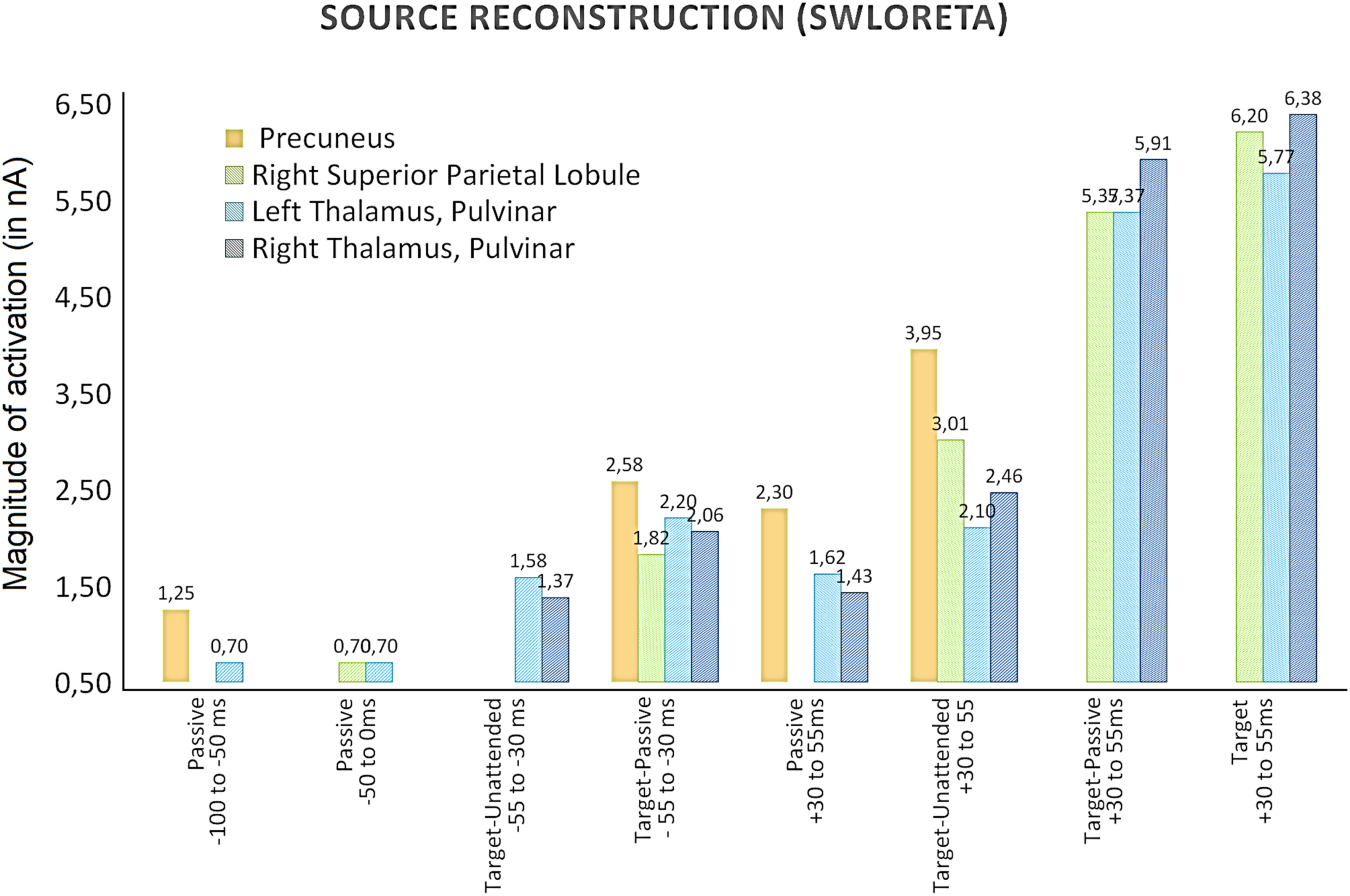
Magnitudes of strength of activation (in nA) of electromagnetic dipoles explaining surface potentials recorded in different pre‐ and post‐stimulus attention condition, according to swLORETA: Here depicted are the amplitude values of signals deriving from precuneus, bilateral thalamus and right superior parietal lobule, as based on inverse solutions

The swLORETA applied to the different brain potentials, both pre‐stimulus and in the N40 latency range, can be inspected in Figure 8, although a list of all significant electromagnetic dipoles obtained is reported in Table 2. It can be observed that before stimulus presentation, electromagnetic signals were extremely weak, or under‐threshold, thus suggesting an optimal signal‐to‐noise ratio. A mild activation of the left and right thalamic nuclei (pulvinar), left precuneus and bilateral parietal cortex was found between −55 and −30 ms for the target‐passive difference potentials (reflecting a tendency for a pre‐stimulus alertness increase). A mild activation was also found for thalamus and especially the right precuneus (BA7/31) in the N40 range in response to passively observed gratings. No activity of the occipital cortex was found before 60 ms. The most powerful sources explaining attention N40 modulation were the left and right thalamic nuclei of pulvinar (for alertness, attention and spatial frequency selection), cingulate cortex (CC) (BA23) especially for attentional selection of spatial frequency (Conditions 7 and 8), the bilateral parietal cortex (BA40), bilateral precentral cortex and insula (see Table 2 for further details). Figure 9 depicts comparatively the electromagnetic responses attributed to thalamus, precuneus and right superior parietal lobule by swLORETA in the different pre‐stimulus and post‐stimulus attention conditions. It is clearly highlighted how thalamic nuclei and the right superior parietal lobule played a crucial role in attentional allocation at N40 latency range. The significant activation of bilateral thalamus for explaining the difference voltages elicited by target‐minus‐unattended spatial frequency gratings (target‐unattended +30–55 ms) also demonstrates the role of thalamus in visual processing and sensory gating.

**TABLE 2:** List of active electromagnetic dipoles (along with their Talairach coordinates) explaining the scalp‐recorded potentials measured in different time windows and attention condition

				Pre‐stimulus				N40 (+30 + 55 ms)				+60–90 ms
				1. Passive −100‐50 ms	2. Passive −50‐0 ms	3. Target‐unat. −55‐30 ms	4. Target‐passive −55‐30 ms	5. Passive	6. Target‐unat.	7. Target‐passive	8. Target	9. Passive
Lobe	H.	Gyrus	BA	Default mode network			Alertness	Vision	Spatial frequency selection	Alertness	Attention	Vision
Sublobar	L	Thalamus, pulvinar		0.75	0.76	1.58	2.20	1.62	2.1	5.37	5.77	‐
Sublobar	R	Thalamus, pulvinar		‐	‐	1.37	2.06	1.43	2.46	5.91	6.38	1.67
F	L	MFG/SFG	9/10	0,74	0.74	0.86	0.61	0.51	‐	1.87	‐	0.55
F	R	MGF/SFG	9/10	‐	0.93	0.72	0.92		0.9	1.97	1.93	‐
Sublobar	R	Insula	13	‐	‐	1.04	1.48		2.41	5.96	6.51	‐
P	L/R	Supramarginal	40		0.58	‐	‐	‐	‐	5.37	6.20	‐
CC	R	Cingulate	23	‐	‐	‐	‐	‐	‐	6.07	6.71	‐
P	L	Precuneus	7/31	1,25	‐	‐	2.58	‐	‐	‐	‐	‐
P	R	Precuneus	7/31	‐	‐	‐	‐	2.30	3.95	‐	‐	‐
P	L	Postcentral	5/3	‐	0.75	‐	1.56	‐	‐	‐	‐	‐
P	R	SPLobule	7	‐	‐	‐	1.82	‐	3.01	‐	‐	‐
T	L	ITG/STG	20/41	1,10	‐	1.42	1.74	1.37	‐	‐	4.83	‐
T	L	MTG	21/22	‐	‐	‐	‐	0.84	‐	4.71	‐	‐
R	O	MOG	19	‐	‐	‐	‐	‐	‐	‐	‐	3.52
L	O	Cuneus	19	‐	‐	‐	‐	‐	‐	‐	‐	3.13
T	R	MTG	21	1.34	1.17	‐	‐	1.49	‐	‐	‐	‐
F	L	Precentral	6	0.99		‐	‐	1.06	‐	‐	‐	‐
F	R	Precentral	6	‐	0.65	‐	‐		‐	3.23	3.74	‐

*Notes*: Different time windows and attention condition, namely, to passive stimuli in the pre‐stimulus −100/−50 ms time window; to passive stimuli between −50 and −0 ms; to the difference signals obtained by subtracting potentials to target minus irrelevant stimuli (close + far) between −55 and −30 ms; to the difference signals obtained by subtracting potentials to target minus passive stimuli between −55 and −30 ms; to passive stimuli in the N40 range (30–55 ms time window); to the difference signals obtained by subtracting potentials to target minus irrelevant stimuli (close + far) in the N40 range (30–55 ms); to the difference signals obtained by subtracting potentials to target minus passive stimuli in the N40 time range (30–55 ms); to target stimuli in the N40 time range (+30–55 ms); to passive stimuli in the 80–100 ms time range The source reconstructions were based on swLORETA technique. The strength of electromagnetic dipoles (magnitude) is expressed in nA (nanoamperes). Shaded areas highlight magnitudes greater than 1.5 nA.

Abbreviations: BA, Brodmann area; CC, cingulate gyrus; FG, fusiform gyrus; H., hemisphere; ITG, inferior temporal gyrus; Magn., magnitude; MFG, medial frontal gyrus; MTG, middle temporal gyrus; MOG, middle occipital gyrus; SFG, superior frontal gyrus; SOG, superior occipital gyrus; SPL, superior parietal lobule; STG, superior temporal gyrus.

The magnitudes of main electromagnetic signals (i.e. thalamic nuclei, precuneus, parietal cortex, insula and CC) recorded across conditions were statistically compared through Kolmogorov–Smirnov tests (*P* < 0.05), providing evidence that brain activation did not differ between the two pre‐baseline passive conditions (1 vs. 2: −100 vs. ‐50 ms; *P* = 0.99). It did not differ between the pre‐stimulus passive (1) and pre‐stimulus target‐unattended (3) contrast (*P* = 0.76); it tended to be greater during the alertness (4) than passive vision (1) in the pre‐stimulus time‐window (*P* = 0.06); it was not statistically greater during the passive condition post‐stimulus (5) versus pre‐stimulus (1: *P* = 0.62). According to Kolmogorov–Smirnov tests, brain activation was much stronger during spatial frequency attentional selection (6: *P* = 0.004), alertness (7: *P* = 0.01) and attention conditions (8: *P* = 0.05), with respect to the passive pre‐stimulus condition (1).

#### 3.2.2 N80 component N80 (60–90 ms)

**FIGURE 10:**
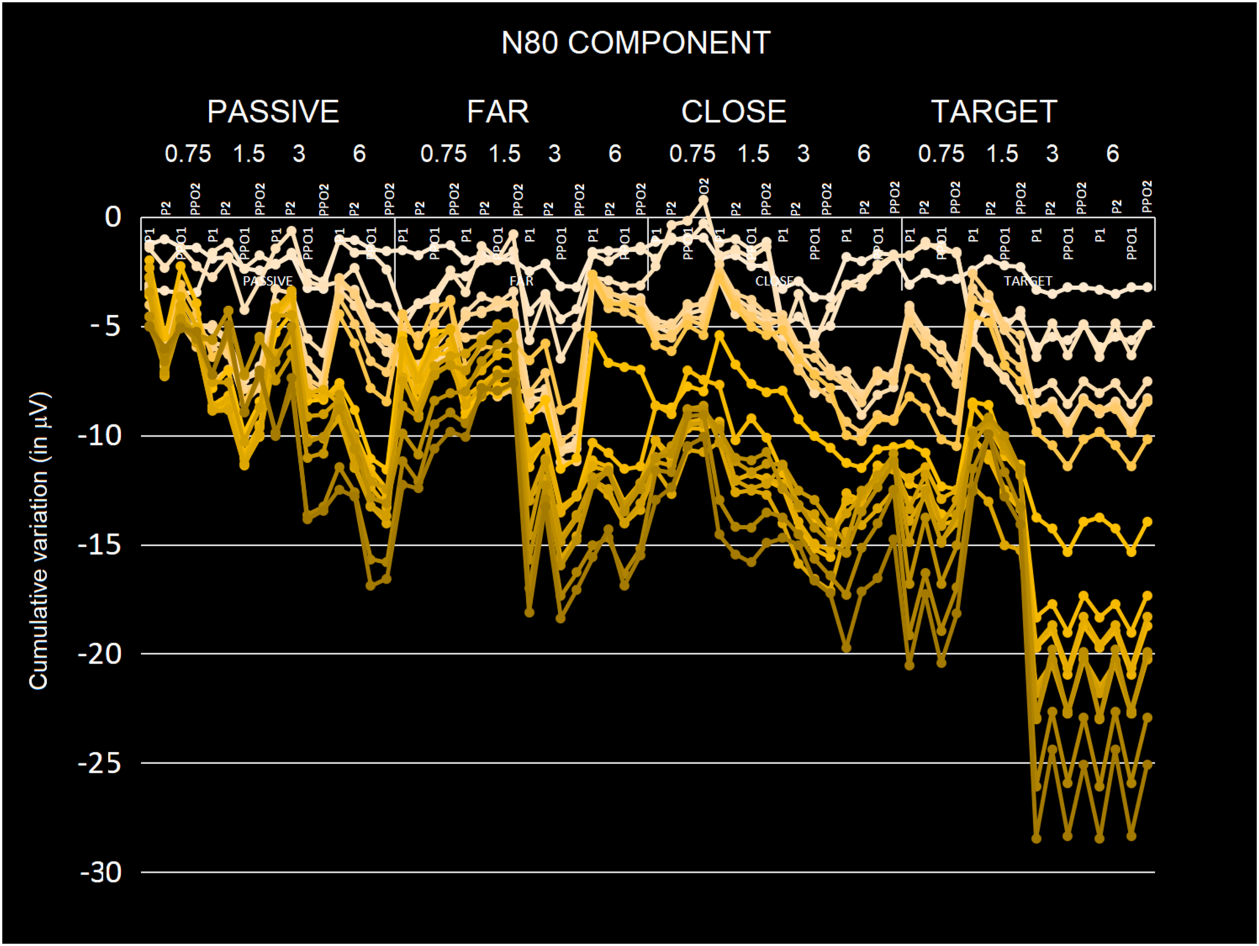
Stacked line charts showing the cumulative variations on N80 amplitude as a function of electrode of recording, cerebral hemisphere, stimulus spatial frequencies and attentional condition. The figure highlights the finer attentional tuning compared with N40 and the stronger sensitivity to high spatial frequency gratings of N80 response. The different traces refer to the different individuals

The ANOVA performed on the amplitude values of N80 response showed the significance of attention factor (*F*(3.54) = 5.07, *P* < 0.0036). Post hoc comparisons showed that the N80 elicited by the attended stimuli (−1.11 μV, SE = 0. 29; [−1.729; −0.495]) was significantly more negative than the N80 elicited by non‐targets (far = −0.68 μV, SE = 0.2, [−1.161; −0.203]; close = −0.78 μV, SE = 0.23, [−1.365; −0.201]) and passively viewed gratings (−0.50 μV, SE = 0.19, [0.911; −0.09]). In turn, N80 to far non‐targets was smaller than that elicited by close non‐targets (see Figure 5, right, and Figure 10).

The significant attention × electrode interaction (*F*(3,54) = 3.87, *P* < 0.0139) and relative post hoc comparisons showed a stronger attentional selectivity at more dorsal than lateral sites. Indeed, whereas all other contrasts among means were significant at PPO1 and PPO2 sites, only at P1 and P2 sites N80 to close non‐targets was different from that elicited by far non‐targets.

The spatial frequency factor was also significant (*F*(3,54) = 3.63, *P* < 0.018); in particular, post hoc tests showed a difference in the N80 response to high versus low spatial frequency gratings. Indeed, N80 response elicited by 6 c/deg gratings (−0.97 μV, SE = 0.2, [−0.911; −0.09]) was significantly more negative compared with the N80 elicited by lower frequencies gratings, namely, 0.75 c/deg (−0.62 μV, SE = 0.23, [−1.09; −0.144]) and 1.5 c/deg (−0.56 μV, SE = 0.2, [−1.12; −0.01]), which were statistically equal among each other. This effect is very well visible in Figure 10. In addition, N80 elicited by 3 c/deg gratings (−0.93 μV, SE = 0.27, [−1.497; −0.35]) was more negative than that elicited by lower frequency gratings but did not differ from that elicited by 6 c/deg gratings (−0.97 μV, SE = 0.3, −95% = −1.460 μV/+95% = −0.482 μV).

The spatial frequency x electrode interaction (*F*(3,54) = 5.02, *P* < 0.0038) and relative post hoc comparisons showed a finer discriminative response to lower spatial frequency at more dorsal (than ventral) electrode sites. In fact, N80 recorded at mesial occipito‐parietal (P1, P2) electrodes was larger to 0.75 c/deg (−0.64 μV, SE = 0.20, [−1.085; −0.213]) than 1.5 c/deg gratings (−0.54 μV, SE = 0.24, [−1.059; −0.025]). In addition, it differed from higher frequencies, being more positive to lower (0.75 deg and 1.5 deg) than higher spatial frequencies, namely, 3 c/deg (−0.86 μV, SE = 0.36, [−1.414; −0.314]) and 6 c/deg (−0.95 μV, SE = 0.22, [−1.43; −0.47]). The same pattern was visible at more ventral sites (PPO1, PPO2) where N80 to 3 c/deg (−0.99 μV, SE = 0.29, [−1.590; −0.382]) and 6 c/deg (−0.99 μV, SE = 0.24, [−1.460; −0.482]) gratings was larger than that elicited by 0.75 c/g (−0.60 μV, SE = 0.24, [−1.493; −0.486]) and 1.5 c/g gratings (−0.59 μV, SE = 0.28, [−1.118; −0.016]).

#### 3.2.3 P1 component (90–120 ms)

**FIGURE 11:**
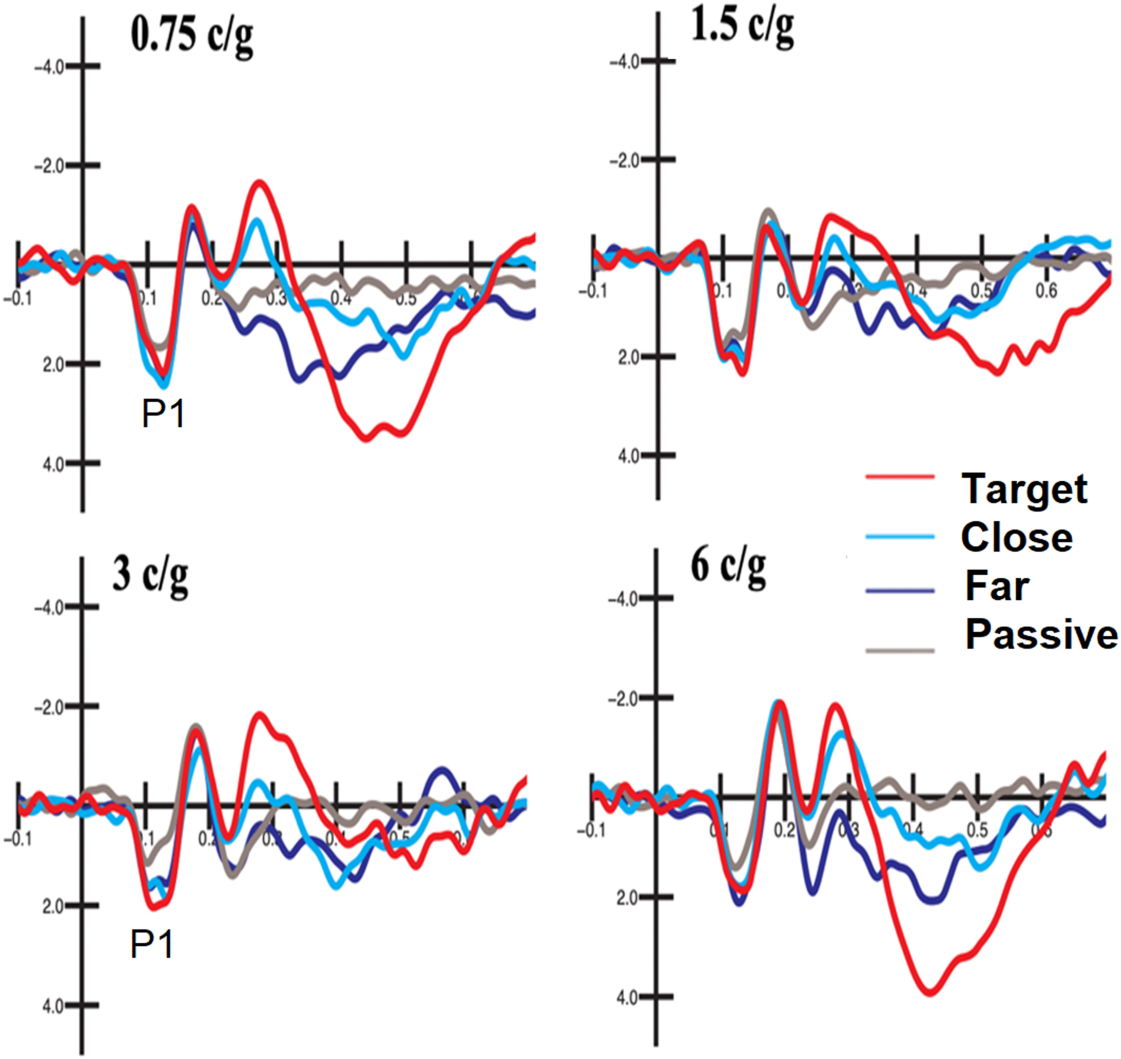
Grand‐average ERP waveforms recorded at a left occipito‐temporal site (P7) in response to gratings of 0.75, 1.5, 3 and 6 c/deg in the different attentional conditions. It is possible to see the clear P1 modulation as a function of alertness (passive vs. attentive conditions)

Figure 11 shows the grand‐average ERP waveforms recorded at a left occipito‐temporal site (P7) in response to gratings of 0.75, 1.5, 3 and 6 c/deg in the different attentional conditions. The ANOVA carried out on the mean area values of P1 response (90–120 ms) showed the significance of spatial frequency (*F*(3,54) = 12.20, *P* < 0.0001). Post hoc comparisons showed that P1 was larger to 0.75 c/g (0.966 μV, SE = 1.96, [−3.16; 5.09]) than 3 c/deg (−0.42 μV, SE = 3.1, [−7.05; 6.207]) and 6 c/deg (−0.‐1.405 μV, SE = 3.1, [−7.93; 5.12]), whereas P1 elicited by 1.5 c/deg gratings (0.44 μV, SE = 2.5, [−4.826; 5.707]) was larger than that elicited by 6 c/deg gratings.

The significant interaction of attention × electrode (*F*(3,54) = 9.48, *P* < 0.0001) and relative post hoc comparisons showed significant alertness effects only at mesial (O1, O2) than lateral (PPO1, PPO2) occipital sites. Post hoc comparisons showed that P1 component to passively watched grating stimuli (−0.24 μV, SE = 2.1, [−4.673; 4.183]) was smaller than to far non‐targets (*P* < 0.01; 0.15 μV, SE = 2.3, [−4.757; 5.058]), close non‐targets (*P* < 0.01; 0.15 μV, SE = 2.2, [−4.490; 4.8]) and target gratings (*P* < 0.001; 0.25 μV, SE = 2.3, [−4.59; 5.086]). Overall, selective attention increased P1 amplitude, but the effects were overall only related to the attentional versus passive viewing contrast. The effect can very well be appreciated by looking at waveforms of Figure 11.

#### 3.2.4 N2 component (250–350 ms, *selection negativity*)

**FIGURE 12:**
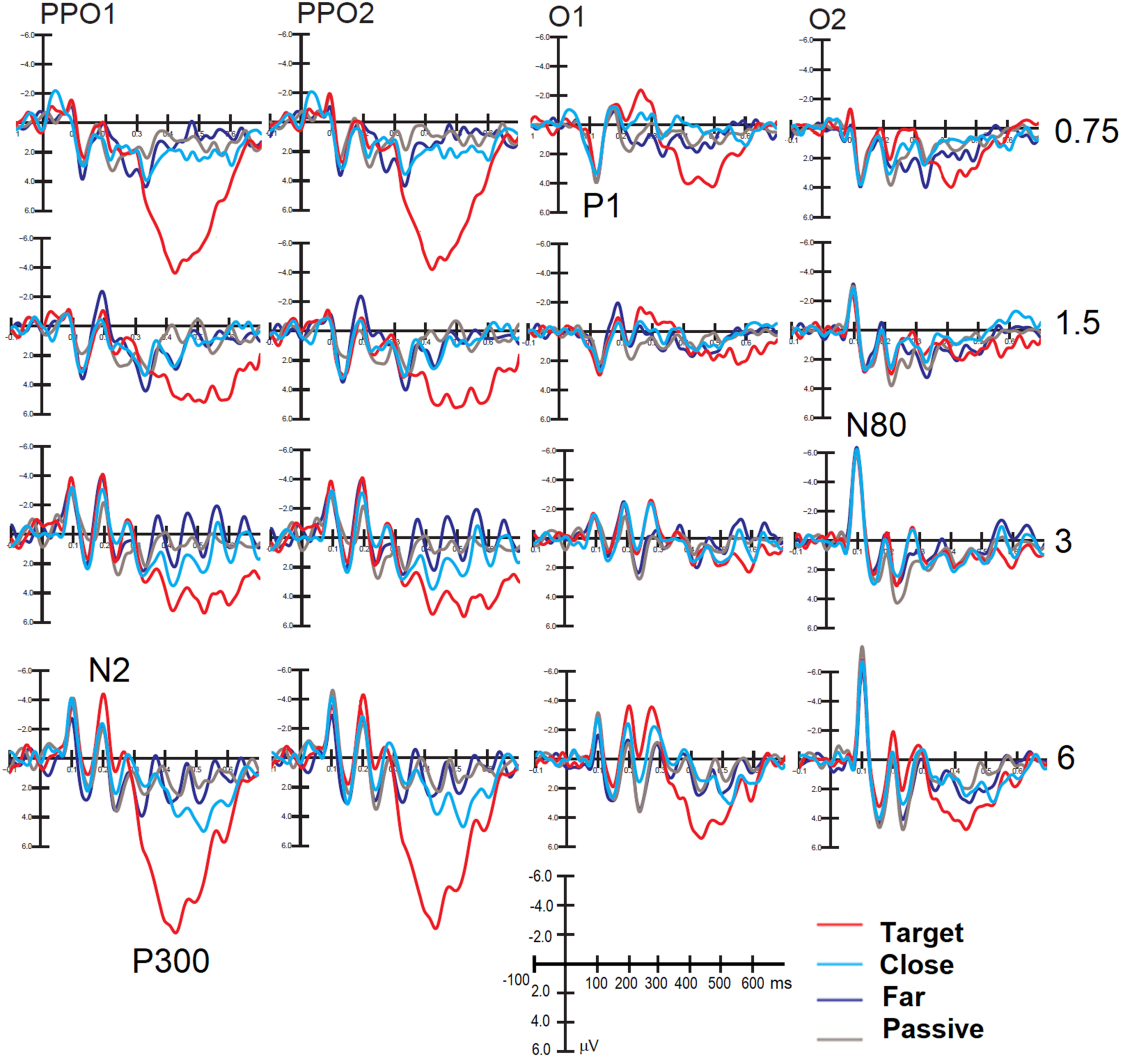
Grand‐average ERP waveforms recorded at a left and right mesial (O1, O2) and lateral (PPO1, PPO2) occipital sites in response to gratings of 0.75, 1.5, 3 and 6 c/deg in the different attentional conditions. It is possible to observe the advantage of extreme frequencies, less subject to interference by non‐target neighbours, in terms of N2 and P300 amplitude. It is also quite visible the nice P/N80 inversion as a function of stimulus spatial frequency, especially at right mesial occipital site

Figure 12 illustrates grand‐average ERP waveforms recorded at left and right mesial (O1, O2) and lateral (PPO1, PPO2) occipital sites in response to gratings of 0.75, 1.5, 3 and 6 c/deg in the different attentional conditions. It is visible the large N2 enhancement and P300 enhancement, especially prominent in response to the easiest targets (0.75 and 6 c/deg), but very significant for all target gratings.

The ANOVA applied to N2 mean area amplitude values showed a significant attention effect (*F*(3,54) = 12.74, *P* < 0.0001). Specifically, post hoc comparisons showed a greater negativity to targets (−0.36 μV, SE = 0.5, [−1.54; 0.82]) and slightly irrelevant stimuli (close = 0.146 μV; SE = 0.47, [−0.831; 1.12]) compared with N2 elicited by strongly irrelevant stimuli (far = 1.09 μV; SE = 0.39, [0.27; 1.919]) and passively observed stimuli (0.77 μV; SE = 0.44, [−0.158; 1.71]).

This effect can be appreciated by looking at waveforms of Figures 11 and 12. The spatial frequency factor was statistically significant (*F*(3,54) = 7.94, *P* < 0.0001). Post hoc contrasts revealed a difference in N2 elicited by high versus low frequencies, where low frequencies, 0.75 c/deg (0.64 μV, SE = 0.40, [−0.916; 1.472]) and 1.5 c/deg (0.62 μV, SE = 0.44, [−0.297; 1.53]), elicited a more positive wave than high frequencies, 3 c/deg (0.24 μV, SE = 0.47, [−0.74; 1.23]) and 6 c/deg (0.15 μV, SE = 0.48, [−0.875; 1.179 μV]).

The attention × frequency electrode interaction was statistically significant (*F*(3,54) = 1.95, *P* < 0.050). Post hoc comparisons showed that N2 was significantly (*P* = 0.009) larger to target than close non‐targets only for to 3 c/deg target gratings, whereas for all spatial frequencies, N2 elicited by targets differed (*P* < 0.0005) from those elicited by far non‐targets (see Figure 11).

#### 3.2.5 P300 component (400–600 ms)

The ANOVA performed on P300 mean area amplitude values showed a significant effect of the attention factor (*F*(3,57) = 56.59, *P* < 0.0001). Post hoc comparisons showed that P300 was larger to targets (10.41 μV, SE = 4.8, [0.35; 20.46]) than close and far non‐targets or passively observed stimuli (close = 6.35 μV, SE = 4.99, [−4.09; 16.79]; far = 5.71 μV, SE = 5.02, [−4.81; 16.22]; passive = 4.93 μV, SE = 5.06, [−5.66; 15.52]); moreover, the P300 was more positive in response to the slightly unattended condition (close) compared with the passive observation condition (see Figures 11 and 12).

The spatial frequency factor was also significant (*F*(3,57) = 7.44, *P* < 0.0001). In particular, post hoc analysis showed that P300 response elicited by 0.75 c/deg gratings (7.31 μV, SE = 4.9, [−3.03; 17.65]) was significantly more positive than that elicited by the intermediate frequencies of 1.5 c/deg (6.56 μV, SE = 4.98, [−3.86; 16.98]) and 3 c/deg (6.48 μV, SE = 4.98, [−3.94; 16.9]), whereas the P300 elicited by 6c/deg gratings (7.04 μV, SE = 4.95, [−3.32; 17.41]) was significantly more positive than that elicited by 3 c/deg and lower frequencies.

## 4 DISCUSSION

The electrophysiological literature reports short‐latency components that mediate sensory perception in the auditory (Adler et al., 1982), somatosensory (Goldring et al., 1970) and visual channels (Kraut et al., 1985). The subcortical visual potentials, however, have remained a subject of debate for many decades, due to the complex anatomy of the visual system and the different stimulation approaches (light pulse, flash, pattern, steady state, etc.). The aim of this investigation was to evaluate whether, on the one hand, with an optimal signal‐to‐noise ratio, the visual N40 to spatial frequency gratings could be detected in the scalp‐recorded waveforms and, on the other hand, whether it was modulated by the sensory and attentional properties of the stimulus.

### 4.1 Scalp‐recorded pattern‐onset N40 component

We identified a negative deflection of about 40 ms in latency, quantified between 30 and 55 ms over the occipital areas (O1, O2, PPO1, PPO2), which was modulated by attention, being more negative for the target than for all other stimuli (non‐targets and passively viewed stimuli). This response probably corresponds to the early VEP response (N40) reported in monkey (e.g. Givre et al., 1994; Kraut et al., 1985, 1990; Schroeder et al., 1991, 1992; Tenke et al., 1993) and human studies (e.g. Harding & Rubinstein, 1980, 1982; Pratt et al., 1982; Pratt et al., 1995; Pratt et al., 2000) thanks to intracranial and scalp recordings. The temporal window (in the late part) is partially overlapping with the early phase of C1 response, as reported, for example, by Proverbio et al. (2010) who found a prominent contribution of BA17 in the scalp‐recorded activity between 40 and 60 ms, but it is conceivable that the arising of C1 may be overlapped with N40 descending. In this study, N40 was not affected by stimulus spatial frequency per se, but only by attention condition. On the contrary, besides being affected by attention, later N80 was instead more negative to high than to low spatial frequencies. The C1 (N80) modulation as a function of stimulus spatial frequency is highly consistent with previous electrophysiological literature (Bodis‐Wollner et al., 1992; Jeffreys & Axford, 1972; Proverbio et al., 1996; Regan, 1989; Zani & Proverbio, 2009) and hints at the striate origin of this response (Dagnelie et al., 1989), which is specialized for the processing of high spatial frequencies of visual information (e.g. Foster et al., 1985).

Because the asymmetrical conformation of the dendritic arborizations of stellate cells has been proofed to allow the generation of electric fields detectable at a distance (Lund, 1973), showing how the EEG technique can actually detect subcortical activity with a certain precision (Seeber et al., 2019), we carried out an analysis of the neural sources of N40 with swLORETA. To identify the neural source of the earliest attentional effect, the source reconstruction was applied to the targets‐minus‐non‐targets difference waves, regardless of stimulus spatial frequency. The strongest sources for N40 attentional modulation were the precuneus (BA7), the superior parietal lobule (BA7), the superior frontal gyrus (BA10), the pulvinar, the insula (BA13) and the thalamus. This pattern of results fits with the literature (for a comprehensive review see Saalmann & Kastner, 2014) showing how attentional allocation is mediated by the fronto‐thalamic‐mesencephalic interconnections, involving the superior frontal gyrus (BA10), the superior parietal lobule (BA7), pulvinar and thalamus. Early sensory modulation appears to be linked to higher order processes mediated by the frontal areas (Johnson & Knight, 2015; Luo & Maunsell, 2018). Similarly, the attentional modulation of N80, P1, N2 and P300 would be the late reflection of top‐down mechanisms from the parietal and frontal regions, which would be engaged in less than 30 ms from stimulus presentation (Banerjee et al., 2019). The modulation of thalamic‐frontal pathways is assumed to reflect ascending attention processes engaged by external sensory inputs (Jagtap & Diwadkar, 2016). The attentional allocation would therefore result in an increase in the amplitude of neural discharge in the visual areas (Buffalo et al., 2010). The present data show that the visual N40 is modulated by attention, possibly reflecting the early activity of thalamic LGN (Bender & Youakim, 2001; Kastner & Pinsk, 2004; McAlonan et al., 2008; Saalmann & Kastner, 2014; Schneider, 2011).

Source reconstruction data from the present study strongly fit with the available neuroimaging literature. Left and right thalamic nuclei were found significantly more active during attentional conditions (nos. 6, 7 and 8) in N40 time range (+30/55 ms) than in passive pre‐stimulus condition. This finding agrees with the notion that visual thalamus functions as an early gatekeeper in controlling attentional response (Halassa & Kastner, 2017; McAlonan et al., 2006; McAlonan et al., 2008; Saalmann & Kastner, 2014; Schmitt et al., 2017; Wimmer et al., 2015).

Furthermore, the evidence that thalamic dipoles were more active during visual processing of targets than of irrelevant gratings (as shown by swLORETA solution applied to the difference waves ‘target minus unattended gratings’) demonstrates the visual nature of N40 evoked potential and fits with previous literature (e.g. O'Connor et al., 2002) showing that not only the thalamus is able to enhance neural responses to attended stimuli (as in our attention conditions no. 7 and 8) but also to inhibit neural responses to unattended stimuli (as in our condition no. 6).

The strongest sources of activation of N40 during attentional conditions were bilateral thalamus, insula, supramarginal gyrus, right superior parietal and right precuneus. These areas are part of a functionally interconnected network for visual attention. In addition, the CC was strongly active only during attentional selection of targets, which fully agrees with its role in selective attention, inhibitory control and conflict resolution (Carter et al., 1998; Casey et al., 2000).

Another interesting piece of evidence from this study is that the precuneus was weakly active during the pre‐stimulus passive condition, along with the media temporal lobe, insula and the medial prefrontal cortex. It is known that the ventral posterior portion of insula is highly interconnected with the posterior CC and the medial temporal lobe (Cauda et al., 2010). These regions are part of the so‐called default mode network (DMN) supporting the passive resting state (Utevsky et al., 2016), which is coincident with the passive pre‐stimulus condition of the present study. Pulvinar nuclei of thalamus are also part of the DMN (e.g. Cunningham et al., 2017) and were also found weakly active during the pre‐stimulus passive condition (*P* = 0.06), but their activation strongly increased during the attention and alertness post‐stimulus response, as indexed by N40 potentials. The clear demarcation between pre‐stimulus and post‐stimulus synchronized activity, in terms of statistical significance, networks and functional properties of signals recorded (see Figure 9), robustly supports the reliability of the present source reconstructions and data interpretation.

As for the issue of whether EEG can detect thalamic activity, large evidences were recently provided. In fact, a recent study with high‐density (256‐channel) scalp EEG recorded simultaneously with intracranial local field potentials from deep brain structures in patients undergoing deep brain stimulation (DBS) demonstrated that EEG source localization is able to sense and properly localize spontaneous alpha activity generated in the thalamus (Michel & Brunet, 2019). Again, Seeber et al. (2019) placed DBS electrodes in centro‐medial thalamus (GTS) and accumbens nuclei providing the unique opportunity to record subcortical activity simultaneously with high‐density (256‐channel) scalp EEG. In this study, a significant correlation between alpha envelopes derived from intracranial and EEG source reconstructed signals was found, thus providing a direct evidence that scalp EEG indeed can sense subcortical signals. In his review, Lopes da Silva (2019) conclusively concluded that subcortical local field potentials (LFPs) can reach the scalp EEG by volume conduction and that high‐resolution EEG scalp recordings (as the present 128‐channel montage) can be used to estimate corresponding sources localized in deep subcortical brain areas. Consistently, Cebolla et al. (2017) using swLORETA source reconstruction (the one used in the present study) found thalamic and cerebellar generators for motor imagery by localizing scalp‐recorded EEG.

### 4.2 N80 and later P1, N2 and P300 attentional modulation

N80 response was more prominent at occipito‐parietal sites where it reached the maximum amplitude, as predicted by current literature (Fu et al., 2009; Proverbio et al., 1996, 2010; Zani & Proverbio, 2012). It also showed a stronger attentional selectivity at more dorsal than lateral sites. It was modulated by selective attention, being more negative to the targets than to the non‐targets and passively viewed gratings. In turn, N80 to far non‐targets was smaller than that elicited by close non‐targets, which indicates a more focused attentional filter, as compared with N40. The attentional modulation of N80 due to object‐based selection has been widely documented (e.g. Capilla et al., 2016; Proverbio et al., 2010; Proverbio, Del Zotto, & Zani, 2007; Zani & Proverbio, 2018; Zhang et al., 2015) and attributed to V1 modulation (e.g. Proverbio et al., 2010; Verghese et al., 2012). N80 response was also greater to high versus low spatial frequency gratings as predicted by a consolidated literature (e.g. Bodis‐Wollner et al., 1992; Kelly et al., 2013; Proverbio et al., 1996; Regan, 1989). The LORETA applied to evoked potentials elicited by passively viewed gratings in the 60–90 ms time window identified the strongest sources for the non‐attentive condition in the occipital cortex, namely, in the middle occipital gyrus and the uncus, which is consistent with previous literature (e.g. Vanni et al., 2001).

P1 (90–120 ms) was focused at lateral occipital sites and revealed to be of larger amplitude to lower than higher spatial frequency gratings, in line with previous literature. For example, Proverbio et al. (1996) recorded sensory VEPs to 1.5, 3, 6 and 12 c/deg gratings finding that whereas low‐frequency patterns elicited a larger positive potential localized at lateral occipital sites, high‐frequency patterns elicited a more prominent midline occipital negative potential, as also showed by scalp current density (SCD) mapping.In this study, P1 was greater to targets than non‐targets and additionally showed significant alertness effects, in that the amplitude of P1 response to passively watched stimuli reached a smaller amplitude than that to irrelevant stimuli. The P1 sensitivity to both object‐based attention (Proverbio et al., 2010; Proverbio, Del Zotto, & Zani, 2007; Zani & Proverbio, 2018) and alertness (Williams et al., 2016; Woldorff et al., 1997; Zani & Proverbio, 2017) is fully consistent with what previously documented.

Again, selective attention to gratings spatial frequency strongly modulated occipito/temporal N2, as predicted by pioneeristic Previc theory of ‘selection negativity’ and in agreement with an extensive ERP literature on object‐based attentional selection (Eimer, 1993, 1997; Harter & Guido, 1980; Harter & Previc, 1978; Kenemans et al., 1993; Luck & Hillyard, 1994; Wijers et al., 1987).

### 4.3 P300 and behavior

The quite speeded average motor RT for the complex discrimination of low‐contrast sinusoidal targets (465 ms) and extremely large‐amplitude P300 component to targets (10.5 μV) indicate that most of participants were very focused and attentive. The results of ANOVA showed a higher accuracy in detecting extreme frequency gratings (i.e. 0.75 and 6 c/deg), which were less affected by stimuli of similar spatial frequency. This was paralleled by larger P300 (and N2) responses to 0.75 and 6 c/deg target gratings. Similarly, RTs were faster to the lowest and the highest frequency patterns. This pattern of results is quite consistent with previous literature showing that the more similar the relevant and irrelevant stimuli, the greater the number of errors (Harter & Previc, 1978; Proverbio, Zani, & Avella, 2007; Zani & Proverbio, 1995). The larger P300 amplitude to the extreme frequencies might therefore indicate a greater ease in discrimination (Patel & Azzam, 2005; Polich, 1997, 2007). P300 component was much larger to target than non‐target stimuli and, in turn, to close irrelevant stimuli than passively viewed stimuli, thus showing an attentional gradient as well as an alertness effects (Justen & Herbert, 2018; Polich, 2007).

## 5 CONCLUSIONS AND FUTURE DIRECTIONS

Overall, the data showed evidence of a scalp recordable pattern‐onset evoked N40 response to spatial frequency gratings. This response was mostly focused over parieto‐occipital areas and was modulated by object‐based selective attention, but not by stimulus spatial frequency per se. This study, in our knowledge, represents the first electrophysiological evidence of an attention effect earlier than P/N80 or C1 component of VEPs/ERPs. Converging neurophysiological and neuroimaging findings suggest that N40 might derive from the EPSP at LGN of thalamus and pulvinar and cortico‐striatal projections. Its activity is thought to be regulated by fronto‐parietal top‐down control, active as early as 30 ms after stimulus onset (Banerjee et al., 2019). These hypotheses are supported by neurophysiological and neuroimaging literature showing how LGN plays a crucial role in attentional control through gating visual sensory signals (McAlonan et al., 2006, 2008; O'Connor et al., 2002; Wimmer et al., 2015) and by amplificating cortical connectivity with the prefrontal cortex (Halassa & Kastner, 2017; Schmitt et al., 2017).

The possible limitations of this study include some residual noise on the ERP waveforms that might be eliminated, in future studies, by increasing the number of ERP trials administered to each subjects (that was already 1800), and especially by eliminating subjects with noisy EEG, procedure that was not applied here to increase data transparency and validity.

As a future direction, it might be interesting to investigate whether abnormal visual N40 response of VEPs might be in future correlated with neural pathologies, as it happens, for example, for auditory and somatosensory N40 responses, which are used as diagnostic cues for schizophrenia (Adler et al., 1982) and hemiparesis from stroke (Peters et al., 2018), respectively.

## CONFLICT OF INTEREST

The authors declare no competing financial interests.

## AUTHOR CONTRIBUTIONS

AMP conceived and planned the experiment. VB prepared the stimuli and carried out the EEG recordings. VB performed statistical analyses and contributed to the interpretation of the results. FDB performed data illustration, plotting and mapping. AMP interpreted the data and took the lead in writing the manuscript. AZ participated to source reconstruction. All authors provided critical feedback and helped shape the research, analysis and manuscript.

### PEER REVIEW

The peer review history for this article is available at https://publons.com/publon/10.1111/ejn.15443.

## Data Availability

Anonymized data and details about preprocessing/analyses are available to colleagues through Figshare platform at https://doi.org/10.6084/m9.figshare.c.5480076.
